# The repeatability and agreement of biometric measurements by dual Scheimpflug device with integrated optical biometer

**DOI:** 10.1038/s41598-022-11953-8

**Published:** 2022-05-11

**Authors:** Hassan Hashemi, Sara Sardari, Abbasali Yekta, Mehdi Khabazkhoob

**Affiliations:** 1 Noor Research Center for Ophthalmic Epidemiology, Noor Eye Hospital, Tehran, Iran; 2 Noor Ophthalmology Research Center, Noor Eye Hospital, Tehran, Iran; 3 Department of Optometry, Mashhad University of Medical Sciences, Mashhad, Iran; 4 Department of Basic Sciences, School of Nursing and Midwifery, Shahid Beheshti University of Medical Sciences, Tehran, Iran

**Keywords:** Biological techniques, Optics and photonics

## Abstract

To determine the repeatability of biometric measurements by dual Scheimpflug Devices with Integrated Optical Biometers and its agreement with partial coherence interferometry according to the axial length (AL), and the presence of cataracts. The present population-based cross-sectional study was conducted on the geriatric population in Tehran. For participants, imaging was performed by dual Scheimpflug Devices with Integrated Optical Biometers (Galilei G6) and partial coherence interferometry (IOL Master 500). All measurements were performed by one person. In both normal and cataractous eyes, the ICC values were above 0.99 for three measurements of AL, intraocular lens (IOL) power target, anterior chamber depth (ACD), central corneal thickness (CCT), flat and steep keratometry readings, and mean total corneal power (MTCP). The repeatability coefficient for the AL measurements was 0.003 and 0.002 in eyes with and without cataracts, respectively. The mean difference of AL between IOL Master 500 and Galilei G6 in normal and cataractous eyes was 0.015 and −0.003 mm, respectively. The 95% limits of agreement (LoA) of AL between these two devices were −0.09 to 0.12 mm in normal and −0.09 to 0.08 mm in cataractous eyes. The 95% LoA of ACD between the two devices was −0.13 to 0.36 mm and −0.10 to 0.31 mm in eyes without and with cataracts, respectively. The 95% LoA of steep K between the two devices was −0.63 to 0.32 and −1.04 to 0.89 diopter in normal and cataractous eyes, respectively. The results of the present study indicate the high repeatability of Galilei G6 in ocular biometric measurements. Galilei biometric measurements, had a very high agreement with the IOL Master 500.

## Introduction

Precise calculation of intraocular lens (IOL) power before cataract surgery is critical to achieving optimal visual outcomes and patient satisfaction, which in turn requires accurate ocular biometry^1–6^. In recent years, advances in IOL technology and surgical methods have made cataract surgery a refractive surgery^5,6^. Also, a significant increase in the number of cataract surgeries in post-refractive eyes and, consequently the need for accurate ocular biometry has caused significant changes and advances in this field^7^. These issues have led to corneal topographic information being considered in addition to conventional axial length (AL) measurements in preoperative cataract surgery assessments^7^. On the other hand, the introduction of new formulas for IOL power calculation such as Olsen and Holladay 2, has led to more attention to other ocular parameters such as the crystalline lens thickness or the horizontal corneal diameter to ensure a more accurate effective lens position^8^. Recently, multi-task instruments have been introduced that, in addition to measuring the AL, provide more accurate and specific information on the topographic conditions of the cornea and other ocular indicators^9–12^. The review of refractive results after cataract surgery in recent years shows that the residual refractive error attributed to the AL measurement error has decreased from about 60 to 17%, indicating the value of recent advances in biometric devices. Galilei is one of these multi-task devices^13,14^. The latest version (Galilei G6), in addition to measuring anterior segment indices, also performs axial length biometry using a light wavelength of 880 nm based on the principle of low coherence reflectomerty^11^. Few studies have been done on the validity and reliability of this device in ocular biometric measurements^11,12,15–22^. The repeatability of Galileo G6 biometric information particularly the AL has received less attention. Most studies have evaluated the agreement of this device with other technologies^15–17,20^. No study has examined the repeatability of the IOL power calculated by Galilei G6. In a study conducted by Jung et al.^16^ to compare the repeatability of Galilei G6 and IOL Master 700 biometric findings, the biometric data of both devices was highly reproducible, but the IOL Master’s repeatability was better than that of Galilei G6. Considering that repeatability is an important and necessary component in confirming the validity and clinical performance of a device^23^, this study aimed to determine the repeatability of Galilei G6 in measuring the AL and the IOL power in different groups with and without cataract.

## Methods

The data of this study were randomly extracted from a population-based cross-sectional study on the geriatric population of Tehran, Iran (Tehran Geriatric Eye Study). The sampling was performed by the multi-stage stratified cluster sampling method. All samples were taken to the examination site free of charge. First, complete demographic, socio-economic, and anthropometric information was collected by the trained person through a face-to-face interview, and then ocular examinations were performed.

The ocular examinations included the measurement of uncorrected and best-corrected visual acuity (UCVA and BCVA) using a LED visual acuity chart (Smart LC 13, Medizs Inc, Korea) at 6 m (m), objective refraction by an auto-refractometer (ARK-510 A, Nidek Co. LTD, Aichi, Japan), subjective refraction, anterior and posterior segment ocular health examination using a slit lamp-biomicroscope (Slit-lamp B900, Haag-Streit AG, Bern, Switzerland) and a + 90 D lens by an ophthalmologist. Exclusion criteria included severe corneal opacity preventing imaging, macular degeneration (due to loss of fixation), BCVA worse than 20/40 and a history of any intraocular surgery.

In the next stage, the participants underwent ocular imaging and biometry using IOL Master 500 (Carl Zeiss Meditec, Jena, Germany) and Galilei G6 Lens Professional (Ziemer Group, Port, Switzerland). The IOL Master 500 is the gold standard of modern optical biometry instruments. The IOL Master 500 uses the partial coherence interferometry (PCI) principle for AL measurements, a six-point telecentric reflectometry technique for keratometric readings, and an image-based slit illumination for anterior chamber depth (ACD) measurements. It can also measure white to white (WTW) distance. However, crystalline lens thickness ^24^ and central corneal thickness (CCT) measurements are not possible with IOLMaster 500. The Galilei G6 is newer optical biometry devise that combines a dual rotating Scheimpflug camera, a Placido disc topographer, and an optical coherence tomography-based A-scan. It performs axial biometry using the light of 880 based on the low coherence reflectometry. The combination of the biometric and anterior segment measurements provides the IOL calculation.

All biometric measurements were performed by one experienced operator and three measurements were made 10 min apart. In this study, cataract classification was performed based on the world health organization (WHO) grading system^25^. Nuclear, cortical, and posterior sub-capsular (PCS) cataracts were defined as having a grade of equal to or greater than 2^25^. AL values less than 22 mm were classified as short, 22 to 24 mm as medium, and more than 24 mm as long^26^. The parameters assessed for Galilei G6 repeatability were AL, mean total corneal power (MTCP), IOL-target (calculated by SRK-T formula), ACD, flat and steep simulated keratometry readings (SimK), CCT, LT, and WTW distance. Also, to evaluate the agreement of this device with IOL Master 500, the AL, ACD, WTW distance, flat and steep K readings were compared.

### Statistical analysis

To evaluate the repeatability of Galilei G6, the mean and standard deviation (SD) of the three measurements were calculated and the Intraclass Correlation coefficient ^20^ was reported. Then, by calculating the within-subjects SD and multiplying it by 2.77, the repeatability coefficient (RC) was reported. The coefficient of variation (CV) was also reported. The closer the RC is to 0, the better the repeatability. To evaluate the agreement of measurements between IOL Master and Galilei G6, the 95% limits of agreement (LoA) and Bland–Altman plots were used^24^. In Bland–Altman plots, the horizontal axis is the average of the studied parameters by two instruments and the vertical axis is the difference of the values between the two devices. The 95% LoA was calculated based on the "SD * 1.96 ± mean difference" between the two devices.

### Ethical issues

This study was approved by the Ethics Committee of National Institute for Medical Research Development affiliated with the Iranian Ministry of Health and Medical Education. The tenets of the Helsinki Declaration were followed and informed consent was obtained from all participants.

## Results

### Repeatability analysis

In this study, 213 eyes of 128 individuals were examined. Of these, 112 eyes (52.6%) belonged to males. The mean age of participants was 53.3 ± 17.6 years (from 21 to 85 years). 52 eyes in this study had cataracts. Mean ± SD values of three measurements for all studied variables are shown in Table 1 along with ICC, RC, and CV. As seen in Table 1, the ICC of three AL measurements was 100% in individuals with and without cataracts. Repeated measures analysis of variance (ANOVA) showed that there was no statistically significant difference between the three AL measurements in eyes with (P = 0.397) and without cataracts (P = 0.666). In normal and cataractous eyes, the RC and CV were better for the AL than other variables. As seen in Table 1, the ICC of AL was 0.995 and 0.987 in eyes with AL < 22 mm with and without cataracts, respectively. However, in eyes with an AL greater than 22 mm whether normal or cataractous, the ICC was 0.999 for AL measurements. The RC and the CV values were close in different groups of AL.

**Table 1:** The mean and standard deviation (SD) of axial length (AL), mean total corneal power (MTCP), intraocular lens power (IOL) target, anterior chamber depth (ACD), flat keratometry (flat-K), Steep keratometery (steep-K), central corneal thickness (CCT), lens thickness (LT) and white-to-white (WTW) in three measurements and repeatability indices in all subjects and according axial length grouping.

		With cataracts						Without cataracts					
		Mean ± SD			ICC	RC	CV	Mean ± SD			ICC	RC	CV
		Time 1	Time 2	Time 3				Time 1	Time 2	Time 3			
All	AL (mm)	23.41 ± 1.03	23.41 ± 1.02	23.41 ± 1.02	1.000	0.003	0.10	23.19 ± 1.07	23.18 ± 1.06	23.18 ± 1.07	1.000	0.002	0.09
	MTCP (diopter)	43.25 ± 1.68	43.27 ± 1.70	43.27 ± 1.71	0.995	0.110	0.31	43.58 ± 1.51	43.59 ± 1.52	43.61 ± 1.55	0.996	0.084	0.31
	IOL-target (diopter)	20.77 ± 2.59	20.78 ± 2.56	20.77 ± 2.55	0.998	0.100	0.72	21.15 ± 2.69	21.18 ± 2.60	21.15 ± 2.69	0.999	0.085	0.73
	ACD (mm)	3.39 ± 0.34	3.38 ± 0.34	3.39 ± 0.34	0.998	0.002	0.57	3.15 ± 0.30	3.15 ± 0.30	3.15 ± 0.30	0.999	0.001	0.48
	Flat-K (diopter)	43.93 ± 1.65	43.91 ± 1.66	43.92 ± 1.66	0.997	0.070	0.25	44.23 ± 1.50	44.24 ± 1.49	44.26 ± 1.52	0.997	0.052	0.27
	Steep-K (diopter)	44.81 ± 1.79	44.83 ± 1.79	44.81 ± 1.75	0.998	0.064	0.26	45.11 ± 1.60	45.09 ± 1.55	45.12 ± 1.55	0.996	0.079	0.30
	CCT (micron)	532 ± 38	532 ± 37	532 ± 37	0.997	14	0.49	527 ± 30	527 ± 32	527 ± 33	0.996	14	0.48
	LT (mm)	3.71 ± 1.10	3.79 ± 0.99	3.74 ± 1.11	0.970	0.271	9.43	4.41 ± 0.30	4.41 ± 0.30	4.41 ± 0.31	0.967	0.023	1.42
	WTW (mm)	11.93 ± 0.39	11.92 ± 0.40	11.95 ± 0.40	0.974	0.032	0.37	11.76 ± 0.38	11.79 ± 0.38	11.79 ± 0.38	0.989	0.013	0.40
AL < 22 mm	AL (mm)	21.70 ± 0.16	21.70 ± 0.16	21.67 ± 0.15	0.987	0.003	0.11	21.32 ± 0.17	21.30 ± 0.17	21.3 ± 0.16	0.995	0.001	0.10
	MTCP (diopter)	45.65 ± 1.81	45.74 ± 1.93	45.71 ± 1.79	0.999	0.039	0.20	46.4 ± 1.51	46.48 ± 1.43	46.55 ± 1.48	0.991	0.146	0.35
	IOL-target (diopter)	24.4 ± 1.78	24.60 ± 2.16	24.49 ± 1.87	0.991	0.077	0.54	24.48 ± 0.75	24.67 ± 0.63	24.69 ± 0.57	0.956	0.149	0.86
	ACD (mm)	2.9 ± 0.40	2.89 ± 0.40	2.89 ± 0.40	0.999	0.001	0.68	2.75 ± 0.39	2.75 ± 0.40	2.75 ± 0.40	0.999	0.001	0.73
	Flat-K (diopter)	46.05 ± 1.49	45.77 ± 1.94	46.06 ± 1.51	0.976	0.527	0.64	47.23 ± 0.92	47.24 ± 0.76	47.30 ± 0.91	0.976	0.118	0.43
	Steep-K (diopter)	46.79 ± 1.64	46.66 ± 1.75	46.79 ± 1.63	0.996	0.089	0.27	48.29 ± 1.27	48.08 ± 1.09	48.00 ± 1.21	0.989	0.159	0.43
	CCT (micron)	524 ± 19	526 ± 17	527 ± 17	0.986	16	0.54	504 ± 12	500 ± 14	499 ± 16	0.990	18	0.64
	LT (mm)	4.51 ± 0.31	4.51 ± 0.34	4.49 ± 0.36	0.993	0.005	0.90	4.73 ± 0.20	4.76 ± 0.19	4.71 ± 0.20	0.959	0.012	1.22
	WTW (mm)	11.48 ± 0.44	11.47 ± 0.44	11.48 ± 0.44	0.999	0.001	0.18	11.28 ± 0.26	11.30 ± 0.26	11.31 ± 0.27	0.991	0.005	0.32
AL 22-24 mm	AL (mm)	23.11 ± 0.53	23.11 ± 0.53	23.11 ± 0.53	0.999	0.003	0.10	23.08 ± 0.47	23.08 ± 0.47	23.07 ± 0.47	0.999	0.002	0.08
	MTCP (diopter)	43.61 ± 1.50	43.64 ± 1.50	43.63 ± 1.54	0.994	0.115	0.32	43.35 ± 1.18	43.39 ± 1.15	43.40 ± 1.16	0.993	0.076	0.30
	IOL-target (diopter)	21.33 ± 1.66	21.30 ± 1.59	21.33 ± 1.57	0.995	0.107	0.72	21.66 ± 1.32	21.63 ± 1.25	21.63 ± 1.28	0.995	0.072	0.65
	ACD (mm)	3.37 ± 0.30	3.36 ± 0.30	3.36 ± 0.29	0.997	0.002	0.61	3.17 ± 0.23	3.17 ± 0.22	3.17 ± 0.22	0.998	0.001	0.44
	Flat-K (diopter)	44.27 ± 1.47	44.29 ± 1.45	44.27 ± 1.47	0.997	0.049	0.23	44 ± 1.13	44.01 ± 1.13	44.04 ± 1.16	0.996	0.046	0.26
	Steep-K (diopter)	45.13 ± 1.67	45.18 ± 1.65	45.14 ± 1.59	0.997	0.074	0.28	44.89 ± 1.20	44.91 ± 1.18	44.94 ± 1.21	0.994	0.067	0.28
	CCT (micron)	533 ± 40	532 ± 39	533 ± 39	0.997	15	0.50	526 ± 32	526 ± 33	526 ± 33	0.998	12	0.42
	LT (mm)	3.80 ± 1.05	3.87 ± 0.93	3.86 ± 1.01	0.965	0.268	8.37	4.38 ± 0.31	4.37 ± 0.29	4.38 ± 0.32	0.959	0.029	1.55
	WTW (mm)	11.91 ± 0.34	11.90 ± 0.35	11.93 ± 0.37	0.969	0.030	0.38	11.8 ± 0.36	11.84 ± 0.37	11.83 ± 0.37	0.992	0.009	0.36
AL > 24 mm	AL (mm)	24.75 ± 0.98	24.73 ± 0.97	24.74 ± 0.96	0.999	0.004	0.10	24.81 ± 0.91	24.79 ± 0.85	24.81 ± 0.90	0.999	0.004	0.11
	MTCP (diopter)	41.78 ± 1.17	41.74 ± 1.14	41.8 ± 1.16	0.989	0.115	0.32	42.78 ± 0.69	42.66 ± 0.81	42.68 ± 0.87	0.985	0.078	0.35
	IOL-target (diopter)	18.02 ± 3.03	18.13 ± 3.06	18.05 ± 3.03	0.999	0.081	0.77	16.95 ± 2.99	17.10 ± 2.73	16.99 ± 3.00	0.999	0.102	0.98
	ACD (mm)	3.56 ± 0.29	3.56 ± 0.29	3.56 ± 0.29	0.999	0.001	0.37	3.41 ± 0.29	3.40 ± 0.30	3.40 ± 0.30	0.998	0.001	0.53
	Flat-K (diopter)	42.43 ± 1.09	42.35 ± 1.09	42.41 ± 1.10	0.996	0.040	0.23	43.36 ± 0.77	43.30 ± 0.73	43.32 ± 0.74	0.992	0.035	0.23
	Steep-K (diopter)	43.39 ± 1.36	43.38 ± 1.38	43.38 ± 1.39	0.998	0.033	0.22	44.09 ± 0.67	44.00 ± 0.77	44.05 ± 0.85	0.982	0.082	0.32
	CCT (micron)	533 ± 37	534 ± 38	533 ± 37	0.998	13	0.43	547 ± 22	548 ± 20	550 ± 21	0.974	20	0.65
	LT (mm)	3.25 ± 1.20	3.36 ± 1.12	3.20 ± 1.33	0.971	0.341	14.74	4.35 ± 0.19	4.35 ± 0.24	4.34 ± 0.20	0.982	0.006	0.98
	WTW (mm)	12.11 ± 0.42	12.10 ± 0.41	12.15 ± 0.38	0.962	0.049	0.38	11.89 ± 0.30	11.93 ± 0.19	11.96 ± 0.15	0.867	0.040	0.64

*ICC* intraclass correlation coefficient, *RC* repeatability coefficient, *CV* coefficient of variation.

The repeatability of the IOL power report showed that the ICC of three measurements was 0.999 and 0.998 in eyes with and without cataracts, respectively. Repeated measures ANOVA showed no statistically significant difference between the three IOL calculations in normal (p = 0.871) and cataractous (p = 0.720) eyes. Also, the RC and CV were not significantly different between the two groups. The repeatability analysis of IOL calculations according to the axial length groups showed that ICC was slightly less in cataractous eyes with AL < 22 mm (ICC = 0.956) compared to the normal eyes as well as cataractous eyes in different AL groups (ICC = at least 0.990). The repeatability in this group (cataractous eyes with AL < 22 mm) was slightly worse than in other groups. The ICC of the three measurements was 0.996 and 0.995 in eyes with and without cataracts, respectively. There was no statistically significant difference between the three measurements of MTCPIOL in eyes with (P = 0.597) and without cataracts (P = 0.688). In cataractous and normal eyes and also all AL groups, the ICC of MTCP measurements was at least 0.985. Regarding other biometric components and according to the ICC index, the repeatability of all indices was very high and acceptable (Table 1). However, the repeatability of the LT, especially in cataractous eyes with an AL of less than 22 mm, was slightly worse than other indices.

### Agreement analysis

**Figure 1:**
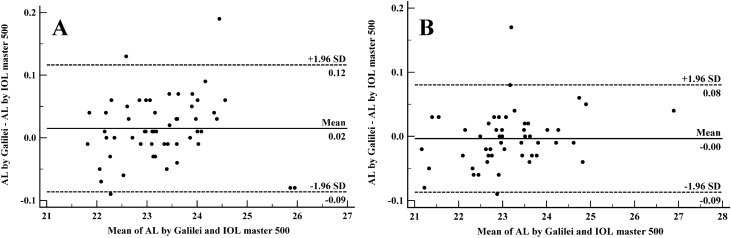
Bland–Altman plots demonstrating 95% limits of agreement between Galilei G6 and IOL master 500 in measuring axial length in normal (**A**) and cataractous (**B**) eyes.

**Figure 2:**
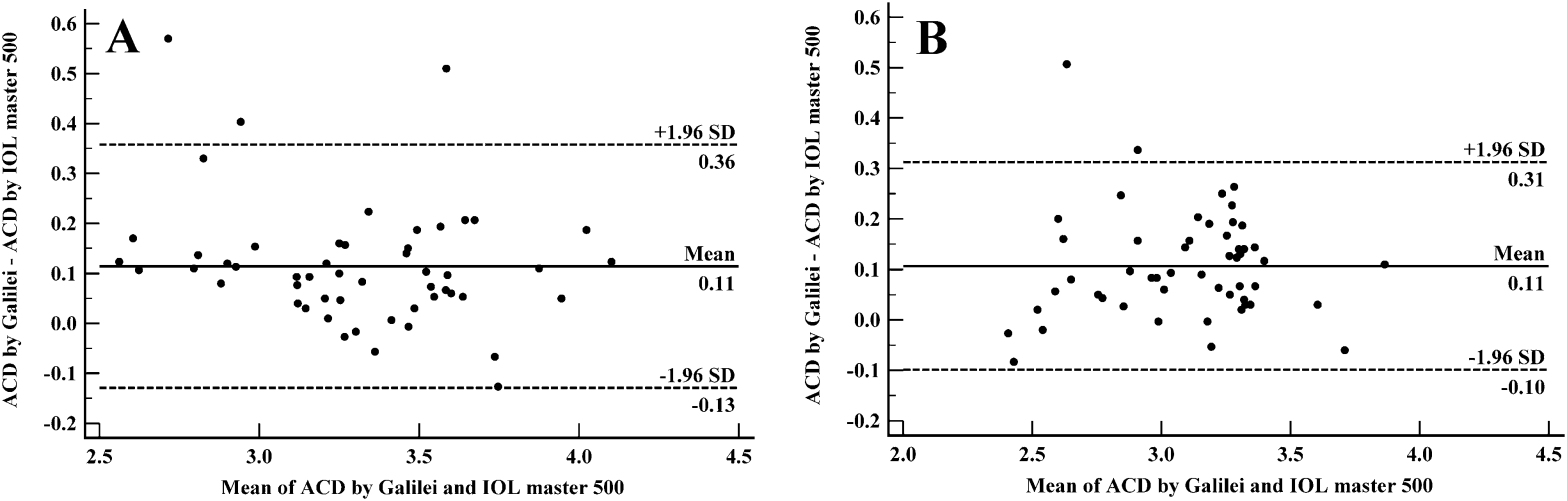
Bland–Altman plots demonstrating 95% limits of agreement between Galilei G6 and IOL master 500 in measuring anterior chamber depth in normal (**A**) and cataractous (**B**) eyes.

**Figure 3:**
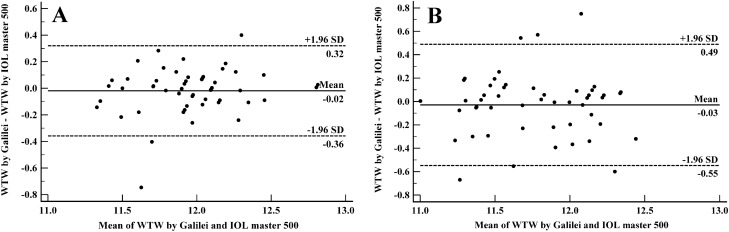
Bland–Altman plots demonstrating 95% limits of agreement between Galilei G6 and IOL master 500 in measuring white-to-white in normal (**A**) and cataractous (**B**) eyes.

**Figure 4:**
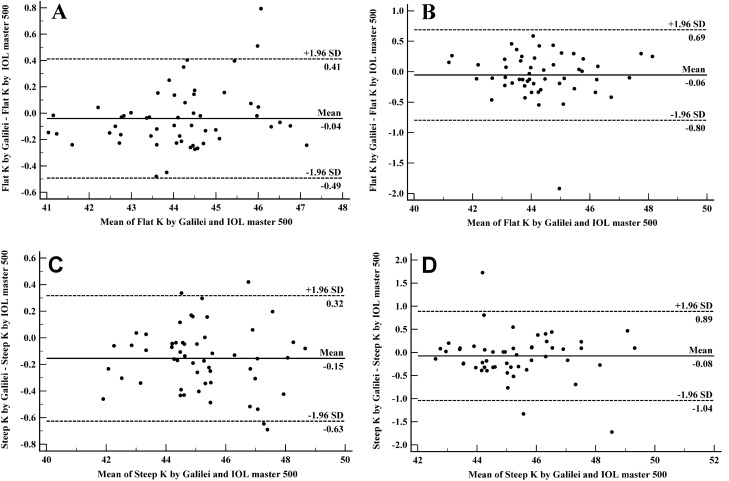
Bland–Altman plots demonstrating 95% limits of agreement between Galilei G6 and IOL master 500 in measuring Flat keratometry in normal (**A**) and cataractous (**B**) eyes and steep keratometry in normal (**C**) and cataractous (**D**) eyes.

Galilei G6 data were compared with IOL Master500 for 106 eyes. Of these, 61 eyes belonged to males, and all cataractous eyes for which repeatability analysis had been performed (52 eyes) were included in this comparison. The comparison of the two devices for some variables is shown in the Table 2. Regarding the AL, the difference between the two devices was 0.015 and −0.003 mm in normal and cataractous eyes, respectively; this difference was statistically significant in normal eyes (P = 0.038). The 95% LoA for AL was −0.09 to 0.12 in normal eyes and −0.09 to −0.08 in eyes with cataracts. Figure 1 shows the Bland–Altman's plots for the agreement of AL between the two instruments separately for normal and cataractous eyes. The ACD obtained by the Galilei G6 was 0.11 mm more in eyes with and without cataracts (P < 0.001). The 95% LoA of ACD was −0.13 to 0.36 and −0.10 to 0.31 mm in eyes with and without cataracts, respectively. Figure 2 shows the Bland–Altman plots for the agreement of ACD between the two instruments in eyes with and without cataracts. The difference of WTW distance between the two devices was 0.02 mm in normal eyes and 0.03 mm in eyes with cataracts, which was not statistically significant in both groups (P values = 0.418 and 0.423, respectively). The Bland–Altman plots for the agreement of WTW distance in normal and cataractous eyes are shown in Fig. 3. As seen in Fig. 3, the 95% LoA of WTW distance was wider in eyes with cataracts. The mean difference of flat K between the two devices was not statistically significant in both eyes with (P = 0.203) and without (P = 0.298) cataracts. Figure 4 illustrates the Bland–Altman plots for the agreement of flat K between the two instruments in normal and cataractous eyes. The 95% LoA of flat K was -0.49 to 0.41 and -0.80 to 0.69 diopter in eyes with and without cataracts, respectively. Regarding steep K, as shown in Table 2, the mean difference between the two devices was significantly higher in non-cataractous eyes (P < 0.001). However, the correlation and agreement of the two devices were better in eyes without cataracts. The proportional error was examined for all Bland–Altman plots and none of them were statistically significant.

**Table 2:** The mean and standard deviation (SD) of axial length (AL), anterior chamber depth (ACD), white-to-white (WTW), flat keratometry (flat-K) and steep keratometery (steep-K) by Galilei G6 and IOL master 500 and their agreement.

		Galileh	IOL master 500	Paired differences	95% LOA	Pearson correlation	p-value
Without cataracts	AL (mm)	23.28 ± 0.89	23.27 ± 0.89	0.015 ± 0.05	−0.09 to 0.12	0.998	0.038
	ACD (mm)	3.37 ± 0.35	3.25 ± 0.38	0.11 ± 0.12	−0.13 to 0.36	0.946	< 0.001
	WTW (mm)	11.94 ± 0.35	11.96 ± 0.32	−0.02 ± 0.17	−0.36 to 0.32	0.869	0.418
	Flat-K (diopter)	44.12 ± 1.43	44.16 ± 1.37	−0.04 ± 0.23	−0.49 to 0.41	0.987	0.203
	Steep-K (diopter)	45.11 ± 1.62	45.27 ± 1.65	−0.15 ± 0.24	−0.63 to 0.32	0.989	< 0.001
With cataracts	AL (mm)	23.14 ± 1.03	23.14 ± 1.02	−0.003 ± 0.04	−0.09 to 0.08	0.999	0.561
	ACD (mm)	3.14 ± 0.33	3.03 ± 0.33	0.11 ± 0.10	−0.10 to 0.31	0.949	< 0.001
	WTW (mm)	11.76 ± 0.39	11.79 ± 0.39	−0.03 ± 0.26	−0.55 to 0.49	0.771	0.423
	Flat-K (diopter)	44.32 ± 1.47	44.38 ± 1.49	−0.06 ± 0.38	−0.80 to 0.69	0.967	0.298
	Steep-K (diopter)	45.26 ± 1.59	45.33 ± 1.63	−0.08 ± 0.49	−1.04 to 0.89	0.954	0.263

*LOA* limits of agreement.

## Discussion

In this study, we attempted to provide a profile of Galilei G6 repeatability as well as its agreement with the IOL Master 500 as a gold standard for optical biometry^27^. The results of the present study showed that Galilei G6 had excellent repeatability in measuring the AL in both individuals with and without cataracts (ICC = 1). A review of the literature found only two studies examining Galilei G6 repeatability^11,16^. Shin et al.^11^ examined the repeatability of Galilei G6 in measuring AL in 25 patients with cataracts and reported ICC and CV values of 0.996 and 0.3, respectively. Jung et al.^16^ compared the repeatability of Galilei G6 with the IOL Master 700 in 101 eyes with cataract. The ICC values for AL measurements were 0.999 and 1 for Galilei G6 and IOL Master 700, respectively, and CV values were 0.147 and 0.021, respectively. The repeatability of Galilei G6 in the present study was slightly better compared to the previous two similar studies. Although factors such as minor measurement errors and demographic characteristics of the samples can contribute to these differences, all three studies indicate the excellent repeatability of Galilei G6 in measuring the AL (all ICC values > 0.90). Pentacam AXL is another multi-task instrument that is capable of anterior segment imaging and biometric measurements simultaneously^9^. Sel et al.^28^ examined the repeatability of Pentacam AXL in measuring AL in 50 eyes of 50 normal adult patients (age range: 18 to 64 years) and reported an ICC value of 0.995. Ruiz-Mesa et al.^29^ performed a similar repeatability study of Pentacam AXL on 80 eyes with and without cataract (40 eyes in each group) and reported ICC values of 0.953 and 0.989 in the two groups, respectively. Based on the results of the present study as well as previous studies, it can be concluded that multi-task topographer/optical biometer systems have excellent repeatability in measuring AL, and in a comparison between these instruments, Galilei G6 has a better repeatability compared to Pentacam AXL.

Based on the results of the present study, the ICC for AL measurements in eyes with an AL of less than 22 mm was lower than in eyes with a longer AL. This finding could be due to the greater impact of repeated measurements on short AL values and has been shown for other biometry devices^30,31^. This finding has also been confirmed for IOL power calculation^32^. Galilei G6’s ICC in measuring LT, although above 0.95, was lower than other indices. The less repeatability of Galilei G6 in measuring LT has already been demonstrated by Jung et al.^16^ and Shin et al.^11^.

The accurate IOL power calculation is the ultimate goal of ocular biometry. In the present report, the repeatability of Galilei G6 in the IOL power calculation was also investigated. So far, no study has evaluated the repeatability of the IOL power calculated by Galilei G6. Because SRK/T is one of the most common formulas for IOL power calculation, we reported the IOL power based on the SRK/T. In this formula, keratometry readings and AL are the main variables. According to the ICC index, the repeatability of Galilei G6 in the IOL power calculation was 0.999 and 0.998 in eyes with and without cataracts, respectively, which indicates this device has excellent repeatability based on the SRK/T formula. This finding is predictable due to the high repeatability of this device in both AL and keratometric measurements. Due to the lower repeatability of Galilei G6 in measuring the ACD based on the present study as well as previous studies, the IOL power calculated with the newer generation formulas that take into account ACD may be less reliable. Given the recent widespread use of these formulas, it seems that this issue needs further investigation and is recommended to be considered in future studies.

In the present study, the agreement of the AL measurements by Galilei G6 and IOL Master 500 was examined. The IOL Master 500 is one of the first generation optical biometers and its validity and reliability have been reported by several studies. According to the results, there was a very high correlation between the ALs measured by Galilei G6 and IOL Master 500 in both normal and cataractous eyes. Also, the 95% LoA between the two devices was narrow and close in the two groups. This finding shows the high agreement of these two instruments in measuring the AL so that they can be considered interchangeable. Galilei G6 uses a newer technology (OLCR vs. PCI) that has better penetration power and higher acquisition rate compared to the IOL Master 500, which can be considered as the advantages of this device. The results of our study are consistent with previous studies on this finding. Jung et al.^16^ showed that in patients with cataracts, Galilei G6 and IOL Master had very similar results, however, the difference between these two devices was more than our study. Shin et al.^11^ compared the Galilei G6 with the Lenstar LS 900. The results of that study showed that the Lenstar’s measured AL was on average 0.04 mm longer than the Galilei G6, which was statistically significant, although the 95% LoA was narrower compared to the present study. Part of this difference can be explained by the shorter wavelength in the Lenstar compared to Galilei G6, although this difference (0.04) does not seem to have clinical significance.

Although our main focus was on the AL, the significant difference in ACD between the two devices in both normal and cataractous eyes was interesting. According to the results, Galilei G6 measured the ACD about 0.11 mm deeper than the IOL Master 500. The results of the Shin et al.^11^ study showed that IOL Master 700 and Galilei G6 differed only 0.04 mm in the measured ACD, which was not statistically significant. Jung et al. also showed that the ACD was not significantly different between Galilei G6 and Lenstar LS 900. Kunert et al.^33^ reported a 0.17 mm difference in the ACD measurements using swept-source and PCI biometry systems. In a study Hua etal.^34^, there was a difference of 0.01 mm in ACD measured by Tomey OA-2000 biometer and IOL Master. It seems that the mean difference of the two devices in the present study is higher than in other studies; this may be due to the different age distribution and measurement technology. However, the agreement between the two devices is acceptable.

In conclusion, the results of the present study indicate the high repeatability of Galilei G6 in ocular biometric measurements. The repeatability of this device in measuring the ACD is less than other indices. Galilei biometric measurements, have a very high agreement with the IOL Master 500.
